# Molecular Detection of Genetic Material of *Toxoplasma gondii* in Goat Blood Samples from Northern Thailand

**DOI:** 10.3390/vetsci12060555

**Published:** 2025-06-05

**Authors:** Pongpisid Koonyosying, Anucha Muenthaisong, Kanokwan Sangkakam, Kanpitcha Nontasaya, Amarin Rittipornlertrak, Boondarika Nambooppha, Nisachon Apinda, Supawadee Maneekesorn, Nattawooti Sthitmatee

**Affiliations:** 1 Laboratory of Veterinary Vaccine and Biological Products, Faculty of Veterinary Medicine, Chiang Mai University, Chiang Mai 50100, Thailand; anucha.m@cmu.ac.th (A.M.); kanokwansangkakam@gmail.com (K.S.); n.nontasaya@gmail.com (K.N.); amarin.r@cmu.ac.th (A.R.); boondarika.n@cmu.ac.th (B.N.); nisachon.a@cmu.ac.th (N.A.); supawadee.man@cmu.ac.th (S.M.); nattawooti.s@cmu.ac.th (N.S.); 2 Office of Research Administration, Chiang Mai University, Chiang Mai 50200, Thailand; 3 Division of Hematology and Oncology, Department of Pediatrics, Faculty of Medicine, Chiang Mai University, Chiang Mai 50200, Thailand

**Keywords:** *Toxoplasma gondii*, goat toxoplasmosis, dihydrofolate reductase thymidylate synthase (*dhfr-ts*), molecular detection, polymerase chain reaction (PCR), goat blood samples

## Abstract

Toxoplasmosis is a significant parasitic zoonosis caused by *Toxoplasma gondii*. Among livestock animals, small ruminants, especially sheep and goats, are the most infected. This infection is a leading cause of abortion, resulting in considerable economic losses for goat breeders. The present study aimed to assess the prevalence of *T. gondii* infection in goats in northern Thailand, with an emphasis on its potential zoonotic transmission to humans. Polymerase chain reaction (PCR) targeting the *T. gondii* dihydrofolate reductase-thymidylate synthase (*Tgdhfr-ts*) gene was employed for molecular detection. This represents the first report of *T. gondii* molecular detection in blood samples from small ruminants in Thailand by PCR. A total of 176 meat goat blood samples were analyzed, yielding an 8.52% (15/176) positivity rate for *T. gondii* DNA. The selected DNA sequences from the positive *T. gondii* DNA displayed a high degree of nucleotide sequence homology with the reference *Tgdhfr-ts* sequence. Phylogenetic analysis revealed a single clade alongside other *T. gondii* strains, showing no differentiation based on genotype. This study contributes to the understanding of *T. gondii* epidemiology and provides a foundation for future strategies to control and manage *T. gondii* transmission in livestock populations.

## Simple Summary

Toxoplasmosis, caused by the parasite *Toxoplasma gondii*, can be transmitted from animals to humans. Sheep and goats are particularly susceptible, resulting in economic losses for farmers. This study examined the presence of the parasite in goats in northern Thailand using an alternative DNA target in the PCR method. Of 176 goats tested, 8.52% were positive. These findings provide valuable insights into the disease’s transmission and support future control efforts.

## 1. Introduction

Toxoplasmosis is a significant parasitic zoonosis caused by *Toxoplasma gondii* [1]. As reported by the Food and Agriculture Organization of the United Nations, *T. gondii* is recognized as the fourth most common cause of foodborne diseases, constituting a major global public health issue [2]. *T. gondii* infection is transmitted to hosts through the ingestion of tissue cysts present in food and water contaminated with oocysts, as well as via transplacental transmission [1,3]. Among livestock animals, small ruminants, especially sheep and goats, are more widely infected by *T. gondii*. Numerous epidemiological studies have demonstrated the presence of antibodies to *T. gondii* in small ruminants worldwide, with seroprevalence rates varying significantly across regions (ranging from 3% to 92%) [4,5] and a global average of approximately 31.78% [6]. Although most infections in small ruminants are asymptomatic, this parasite is a major cause of reproductive failure in goats, including abortion and stillbirth. Fetal abortion was evidenced by a 35% prevalence in Bangladesh [7], whereas the presence of the parasite in fetal tissues causes economic losses for goat production [3,8,9]. *T. gondii* infection not only causes substantial reproductive and economic losses but also poses public health risks, as the consumption of infected meat or milk can facilitate zoonotic transmission [10].

The epidemiology of toxoplasmosis in goats has not been extensively reported in Thailand. Although previous studies have demonstrated significant exposure to *T. gondii* in several provinces, including Satun and Kanchanaburi [10,11], the disease remains inadequately studied in northern Thailand. Despite the growing goat populations in key provinces such as Chiang Mai, Chiang Rai, and Lamphun, where contiguous areas exist, there is a notable absence of molecular surveillance data for *T. gondii* in this region. While *T. gondii* has been detected in various hosts in other regions of Thailand such as in beef cattle in Kanchanaburi, Ratchaburi, and Nakhon Pathom provinces [12,13], dairy cattle in western Thailand [14], and cats in Khon Kaen province [15], there remains a lack of data on *T. gondii* infection in animal populations in northern Thailand. Additionally, human seroprevalence has been documented among HIV-infected patients in southern Thailand [16].

The serological prevalence of *T. gondii* in animals has been examined worldwide [4]. A positive serological result merely indicates exposure to *T. gondii*, whereas direct detection of the parasite in blood or other clinical samples definitively confirms its presence. Thereby, the establishment of a molecular method for primary, reactivated, or chronic toxoplasmosis diagnosis can detect the presence of circulating parasites, which is useful in such situations [17]. Molecular techniques based on genomic DNA amplification are currently the diagnostic methods used to detect potential *T. gondii* infections in small ruminants. The sensitivity of PCR-based techniques is influenced by the copy number of the amplified *T. gondii* genes, including glycerol-3-phosphate dehydrogenase (*B1*) [18], the genes encoding small subunit ribosomal RNA such as the non-coding 529 bp repeated (REP529) sequence (200–300 copies) [19], ribosomal DNA (110 copies) [20], and the internal transcribed spacer (ITS1) [21]. In addition, other single-copy genes such as the p30 (sag1) major surface antigen [22] and granule-dense antigen gra7 [23] have been applied as molecular targets. However, these gene targets were used in two consecutive PCR reactions, which increased the cost, extended processing time, and raised the risk of contamination.

Dihydrofolate reductase thymidylate synthase (DHFR-TS) is an essential enzyme in the folate pathway, which involves the DNA synthesis of all organisms. This enzyme can be targeted against several infectious diseases [24]. In protozoal parasites, dihydrofolate reductase (DHFR) and thymidylate synthase (TS) are expressed as a bifunctional enzyme (DHFR-TS), while they are separately expressed in other organisms [25]. Several recent studies on *Toxoplasma gondii* dihydrofolate reductase-thymidylate synthase (*Tgdhfr-ts*) have identified novel inhibitors as a potential drug design target focusing on toxoplasmosis treatment [25,26,27]. The DHFR-TS gene can be effectively targeted for PCR amplification using specific primers, as demonstrated in previous studies [28,29,30]. The design of primers is crucial for achieving high sensitivity and specificity in detecting polymorphisms within the DHFR-TS gene, particularly in the context of drug resistance in pathogens such as *Plasmodium falciparum*. Therefore, the dihydrofolate reductase thymidylate synthase gene may be applied as a suitable target for polymerase chain reaction (PCR)-based detection of *T. gondii* in small ruminants.

Hence, this study addresses these critical knowledge gaps by applying a PCR-based approach using in-house species-specific primers targeting the *Tgdhfr* gene to detect *T. gondii* genetic material in goat blood samples. This molecular tool enables accurate assessment of the prevalence of *T. gondii* infection in goats, an important livestock species in the region. The findings aim to provide essential data to inform public health policies and improve livestock management strategies.

## 2. Materials and Methods

### 2.1. Blood Collection

A total of 176 meat goats were randomly selected from goat farms with high population densities across three contiguous provinces in northern Thailand: Chiang Mai (*n* = 62), Chiang Rai (*n* = 82), and Lamphun (*n* = 32), during the period from November 2020 to February 2021. The sample size was determined correspond to population of goats using EpiInfo™ version 7.2.5.0 employing the population survey method, with a confidence level of 80%, an expected frequency of 50%, an acceptable margin of error of 5%, a design effect, and a cluster value set to 1 (Table 1). Permission was obtained from farm owners prior to sample collection. The sampling protocol was approved by the Animal Care and Use Committee at the Faculty of Veterinary Medicine, Chiang Mai University (approval no. S26/2563). Blood samples were collected from jugular veins and immediately transferred into EDTA-K2 lyophilized vacuum blood collection tubes (BD Vacutainer ^®^, Franklin Lakes, NJ, USA). The tubes were kept in a cooled box with ice packs during transport to the Faculty of Veterinary Medicine, Chiang Mai University, and processed immediately upon arrival.

### 2.2. DNA Extraction

Genomic DNA was extracted from approximately 200 μL of each blood sample using the PureLink^TM^ Genomic DNA Mini Kit (Invitrogen, Thermo Fisher Scientific, Waltham, MA, USA) following the manufacturer’s instructions. The extracted DNA was eluted in 50 μL of elution buffer. DNA concentration and purity were assessed using a UV/Vis spectrophotometer DU 730 (Beckman Coulter, Brea, CA, USA). The DNA samples were stored at −20 °C until further use.

### 2.3. PCR Amplification

Amplification of the *Tgdhfr-ts* gene was performed using in-house sensitive species-specific primers designed based on the *Tgdhfr-ts* gene sequence from the pL0017 plasmid (MRA-786) as illustrated in Supplemental Figure S1. The sequences of primers were as *Tg*DT-F (5′tataagcttatgcataaaccggtgtgtctggtc 3′) and *Tg*DT-R (5′ gccagcgcggccgcctagacagccatctccatct 3′). PCR was carried out in a final volume of 30 μL, consisting of 5 μL of DNA template (concentration: 40–200 ng/μL), 15 μL of 2X MyTaq HS Red Mix (Meridian Bioscience, Bioline, Memphis, TN, USA), and 1 μL of 10 μM of each primer, with the remaining volume made up with deionized water. The PCR cycling conditions were as follows: an initial denaturation at 95 °C for 1 min, followed by 35 cycles of denaturation at 95 °C for 15 s, annealing at 50 °C for 1 min, and extension at 72 °C for 10 s. Both negative and positive controls were included in all reactions. *T. gondii* DNA from the pL0017 plasmid (MRA-786), which carries the *Tgdhfr-ts* gene, was used as the positive control. DNA from other goat blood parasites, including *Theileria luwenshuni* and *Babesia ovis*, served as negative controls to confirm the specificity of the primers and the absence of cross-reactivity. Five microliters of each PCR product was analyzed by electrophoresis on a 1% agarose gel in 1X TAE buffer. Gels were stained with Maestro Safe Nucleic Acid Prestained (MR-031203, MaestroGen, Hsinchu City, Taiwan) and visualized under a UV transilluminator.

### 2.4. DNA Sequencing and Phylogenetic Analysis

Two positive samples were randomly selected for DNA sequencing. The PCR reaction for DNA sequencing utilized the *TgDT*-F and *TgDT*-R primers. PCR products were purified using the PureLink™ Quick PCR Purification Kit (Invitrogen, Thermo Fisher Scientific, Waltham, MA, USA). The purified PCR samples, along with the sense primer (TgDT-F) and antisense primer (TgDT-R), were sent to ATGC Co. Ltd. (Thailand Science Park, Thailand). The *TgDT*-F was utilized as a sense primer in nucleotide sequence PP824811 (TgDT_goat113) and PP824812 (TgDT_goat125). In parallel, *TgDT*-R was utilized as an antisense primer in nucleotide sequence PP824813 (TgDT_goat113-RC) and PP824814 (TgDT_goat125-RC). Nucleotide alignments were analyzed by comparing them with the *Tgdhfr-ts* sequence on the pL0017 plasmid (MRA-786) using CLC Sequence Viewer 8 software (QIAGEN Aarhus A/S, Denmark). A phylogenetic tree based on the *dhfr* gene of *Toxoplasma gondii* was constructed using the maximum-likelihood method implemented in MEGA11 software with the best-fit substitution model. Bootstrap values were calculated from 1000 replicates to assess the reliability of the clusters, employing the JC + G + I model. The sequences were compared with various *T. gondii* sequences based on the *Tgdhfr-ts* gene reported in the GenBank™ and Toxodb database, with the DHFR-TS sequence of the goat blood parasite *Babesia bigemina* used as an outgroup.

### 2.5. Statistical Analysis

Descriptive statistics were employed to assess the prevalence of *T. gondii*. The molecular prevalence of infection was calculated based on the proportion of positive PCR results relative to the total number of samples tested. The Chi-square with Yates’ correction test was applied to examine variations in prevalence across different age groups and sexes of goats. Statistical significance was determined at *p*-value < 0.05 using GraphPad Prism version 10.2.3.

## 3. Results

### 3.1. Demonstration of Tgdhfr-ts Gene-Based for PCR Detection of Toxoplasma gondii in Goat Blood Samples

**Figure 1:**
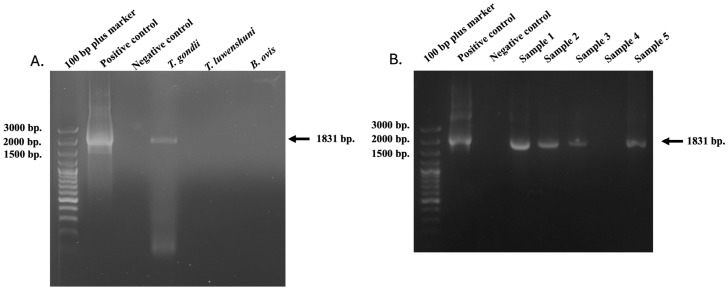
PCR product of *Tgdhfr-ts* gene (1831 bp) amplified from infected goats with *T. gondii* (**A**). compared to other positive samples for goat blood parasites. (**B**). *T. gondii* DNA based on the *dhfr-ts* gene was detected with the conventional PCR in goat blood samples. The molecular size standard is a 100 bp plus ladder, and the tested samples, positive and negative control DNA, were also indicated.

The *Tgdhfr-ts* sensitive species-specific primers were specific for *Toxoplasma gondii* detection only. In contrast, when DNA templates from other goat blood parasites, such as *Theileria luwenshuni* and *Babesia ovis*, were used and amplified with the *Tgdhfr-ts* specific primers, the *dhfr-ts* gene was not detected. This PCR method exhibited no cross-activity when compared under identical conditions (Figure 1A).

### 3.2. Identification of Tgdhfr-ts in the Goat Blood Samples

**Figure 2:**
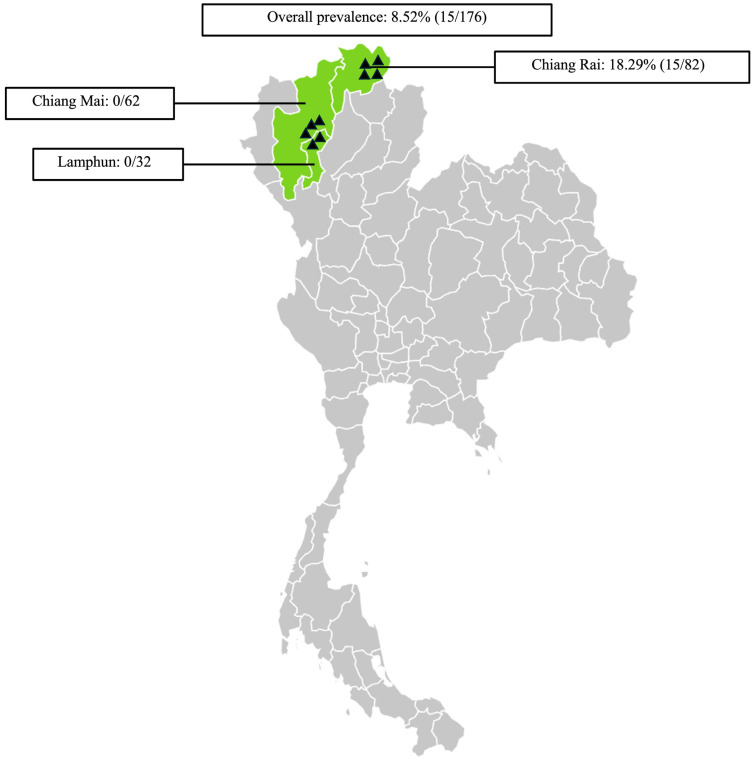
Sampling areas map in northern Thailand associated with the prevalence of *Toxoplasma gondii* infection in goats. ▲ Indicates the locations of the sampled farms. The map was generated using an online infographic tool for geographic visualization (https://create.piktochart.com).

The *Tgdhfr-ts* gene represents a promising target for detecting *T. gondii* parasites through amplification using *Tgdhfr-ts*-specific primers designed based on the *Tgdhfr-ts* gene on the pL0017 plasmid (MRA-786). *T. gondii* DNA was detected with the conventional PCR by the 1831 bp fragment size (Figure 1B). In the three northern Thailand provinces examined, molecular detection of *T. gondii* was found in 18.29% (15/82) of samples from Chiang Rai, while no cases were detected in Chiang Mai and Lamphun. In total, 15 of 176 samples presented positive for *T. gondii*, resulting in an overall prevalence of 8.52% (15/176) (Figure 2). Additionally, statistical analysis revealed no significant differences in the *T. gondii* infection rate between goats older than 2 years (33.33%) and those younger than 2 years (7.18%). Regarding sex, 15 *T. gondii*-positive samples were identified in 165 female samples (9.09%), while no positive samples were found in male goats (0/11). These findings suggest no significant association between sex and *T. gondii* infection, as shown in Table 2.

### 3.3. DNA Sequence and Phylogenetic Analysis

**Figure 3:**
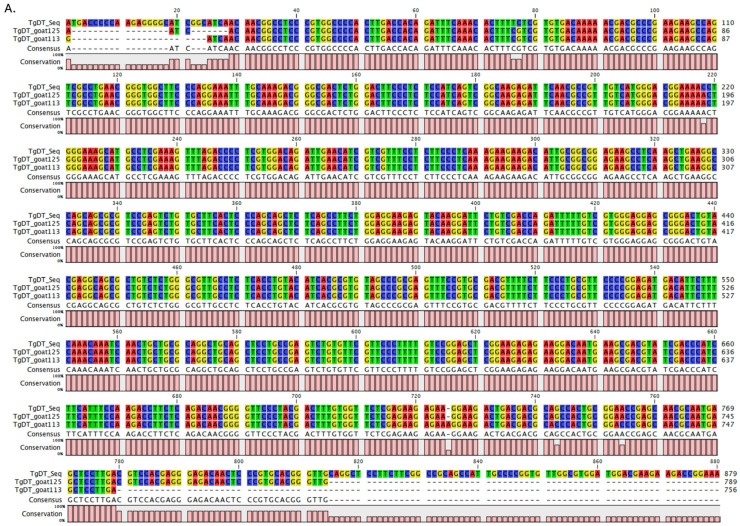

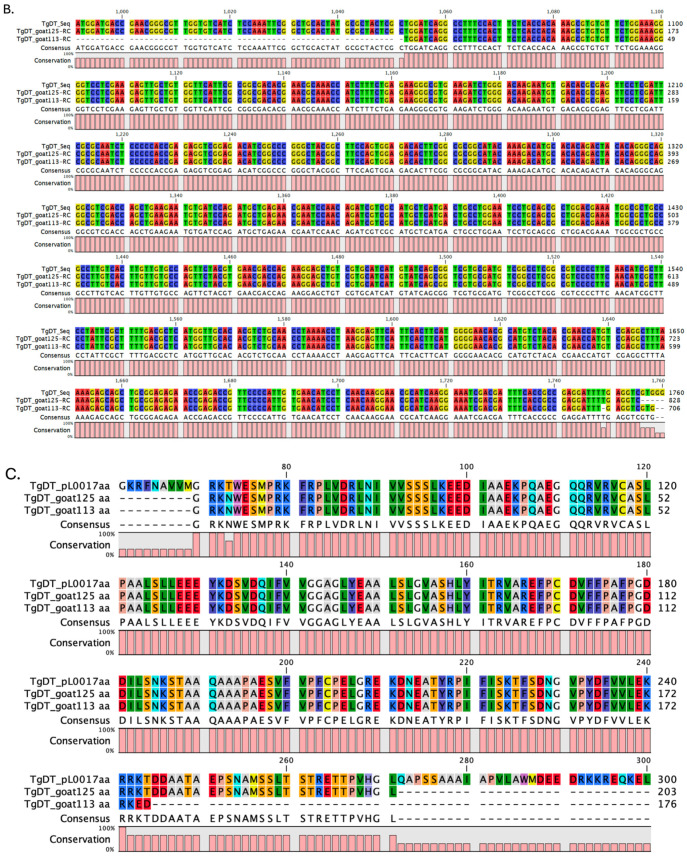
Multiple sequence alignments of the *Tgdhfr-ts* gene between the database and infected goat blood samples: (**A**). The nucleotide alignment using the *Tg*DT-F primer in the sequencing reaction. (**B**). The reverse complement alignment using the *Tg*DT-R primer in the sequencing reaction. (**C**). Protein sequencing analysis in comparison with the *Tgdhfr-ts* sequencing database.

**Figure 4:**
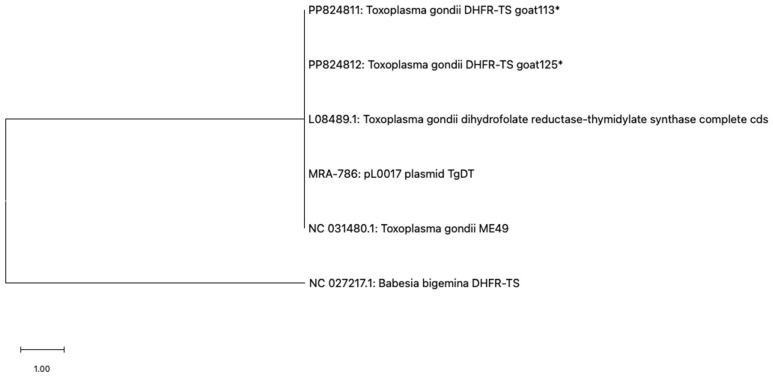
Phylogenetic analysis based on the PCR-amplified *dhfr-ts* sequence of *Toxoplasma gondii* was conducted using the maximum likelihood method with the JC + G + I model. Evolutionary analyses were conducted using MEGA11, with the *T. gondii* sequences (marked with an asterisk) determined in this study and *Babesia bigemina* DHFR-TS used as the outgroup.

Two randomly selected DNA sequences were compared with the reference *Tgdhfr-ts* sequence on the pL0017 plasmid (MRA-786), revealing a high nucleotide sequence homology, as depicted in Figure 3A,B. Furthermore, these nucleotide sequences can be translated into a protein within the reading frame, exhibiting a high degree of homology when compared to the *Tgdhfr-ts* sequencing database (Figure 3C). Phylogenetic analysis revealed that both sequences clustered together with other *T. gondii* strains, showing no differentiation based on genotype (Figure 4).

## 4. Discussion

As far as current knowledge indicates, *Toxoplasma gondii* is a globally distributed protozoan parasite capable of infecting a wide range of animals and humans [31]. Molecular techniques to diagnose toxoplasmosis in small ruminants based on nucleic acid amplification have been reported in various studies [32]. The glycerol-3-phosphate dehydrogenase (*B1* gene) [18] is a ubiquitous PCR target. This study identifies *T. gondii* infection in blood samples obtained from small ruminants in Thailand using PCR targeting specific genes. We focused on detecting the presence of *T. gondii* specifically in goats, which are known intermediate hosts for the parasite. The *dhfr-ts* gene, a well-characterized genetic marker within the *T. gondii* genome, can be amplified and detected by PCR. This specific gene sequence can be amplified within the first run with the amplicon size around 1.8 Kb. PCR amplification of the *dhfr-ts* gene sequences allows the accurate identification of *T. gondii* DNA in goat blood samples. The *Tgdhfr-ts* gene was not detected in other blood parasite strains, confirming that this PCR method exhibited no cross-reactivity. This is particularly relevant for molecular detection, the *dhfr-ts* gene is highly conserved across *T. gondii* strains and plays an essential role in the parasite’s survival and replication [24,25]. Therefore, the *T. gondii dhfr-ts* gene-based PCR can serve as an alternative target for detecting *T. gondii* infection in small ruminants. This method is valuable for identifying the parasite in animal hosts and represents an important advancement in veterinary and parasitology research. Based on our findings from molecular analysis, the prevalence in goats is consistent with reports from Algeria (18.68%), Israel (11.6%), and the Republic of Korea (14.1%) [33,34,35]. A previous study conducted in Iran reported a 1.26% prevalence of *T. gondii* infection, highlighting that diagnostic outcomes based on blood samples may vary. It was noted that the timing of blood infection and its persistence were significantly influenced by the infection with tachyzoites, bradyzoites, and sporozoites. Especially, the tachyzoite stage represents the acute phase of infection [36]. Furthermore, it was observed that older goats had a higher infection rate compared to those under 1 year of age (16.67%). Specifically, the infection rates were 6.45% for goats aged 1–2 years and 33.33% for goats older than 2 years. Consistent with a previous study in China [37], goats aged one year or older showed a higher prevalence of *T. gondii* infection compared to those younger than one year. This suggests that goats acquire *T. gondii* infection through the ingestion of infective oocysts from the environment as they age. However, their sex does not have significant relevance to the infection rate regarding the environment and other factors. The detection of *T. gondii* DNA in 8.52% of goats suggests potential deficiencies in farm biosecurity practices. Although this prevalence is lower than seroprevalence rates reported in other regions, it still indicates active transmission and ongoing exposure risks. Strengthening farm biosecurity, particularly by limiting the access of unauthorized individuals and other animals, and improving feed and water hygiene, is essential for reducing *T. gondii* transmission. The observed prevalence may also be attributed to factors related to intensive farming systems, such as breeding practices, feeding methods, sources of animal water supply, and geographical variation. Notably, oocysts survive longer under cool and moist conditions, which may enhance transmission potential in certain climates. Such variables must be considered when comparing infection rates across geographic areas and study designs [38]. However, in this study, *T. gondii* DNA was detected in 8.52% of goat blood samples using a PCR assay targeting the *Tgdhfr-ts* gene. This detection rate is considerably lower than previously reported seroprevalence rates in goats from other regions of Thailand, which reported rates of 27.9% and 28.5% [10,11]. This discrepancy may be attributed to a positive serological result indicating the infection and immune response, whereas direct measurement of *T. gondii* in blood or other clinical samples presents parasitemia and the diagnosis of primary, reactivated, or chronic toxoplasmosis. Furthermore, this discrepancy can be attributed to the differing diagnostic targets of the two methods. PCR detects active or recent infections by amplifying parasite DNA during the parasitic phase, which is typically transient. In contrast, serological assays detect antibodies that reflect cumulative exposure, often persisting for months or years after infection. The relatively low molecular detection rate likely reflects the brief window during which the parasite circulates in the blood. Once the acute phase subsides, *T. gondii* encysts in tissues, rendering blood-based PCR less effective [17]. In addition, sample type is critical; molecular detection from tissues generally yields higher sensitivity than blood [39]. Despite its lower sensitivity for detecting chronic infections, PCR provides direct evidence of parasite presence and confirms the risk of ongoing transmission within the population. Thus, combining serological and molecular methods in future studies would provide a more comprehensive understanding of *T. gondii* epidemiology in goats, supporting the development of targeted control strategies [40].

Furthermore, the sensitive *T. gondii*-specific sense and antisense primers can be used as a primer in DNA sequencing reactions. The primer sequences have a short open reading frame and consensus sequences with continuous starting and ending. These consensus sequences reveal the *dhfr-ts* gene. It plays a crucial role in various types of sequence analysis, from sequence assembly to profile-based iterative search methods [41]. Although one of the sampled sequences exhibits a mutation compared to the reference sequence, the encoded protein may still be functional. In addition, the deletions within the gene sequences do not affect protein translation. However, these sequencing errors could potentially contribute to anti-DHFR drug resistance, necessitating further in-depth study in the future. Based on the phylogenetic analysis of the *Tgdhfr-ts* gene, the detected sequences in this study were clustered within the same clade, resulting in an effective standardized method for treating and protecting against *T. gondii* infection. A limitation of this study is the limited genotype database available. Accordingly, the PCR-based method targeting the *Tgdhfr-ts* gene, as used in this study, demonstrates high specificity and is suitable for confirming active infections. Although it may not be appropriate for routine surveillance due to cost and infrastructure requirements, it is a valuable tool for early diagnosis in suspected outbreak scenarios or in high-risk populations. Further studies are necessary to identify other species of *T. gondii* infections, determine the specific genotypes of *T. gondii* circulating in Thailand, and explore the potential association between pathogenicity and genotype. Importantly, this finding also raises public health awareness regarding the potential for zoonotic transmission, particularly through the consumption of undercooked goat meat or unpasteurized goat milk, which are consumed in certain regions of Thailand. Given that *T. gondii* tissue cysts can persist in meat and oocysts may contaminate milk, the detection of active infections in goats underscores the need for public awareness campaigns, safe food handling practices, and consideration of pasteurization standards.

## 5. Conclusions

This study demonstrates the applicability of a targeted molecular approach for the detection of genetic material of *T. gondii* in goats, contributing to early diagnosis and improved understanding of the parasite’s prevalence and genetic characteristics within local goat populations. These findings may inform future research on transmission patterns and support the development of targeted control strategies to reduce the risk of spread to other animals and humans.

## Figures and Tables

**Table 1:** Population of goats corresponding to the sample examined.

Province	Population of Meat Goats ^a^			Sample Examined (%)
	Male	Female	Total	
Chiang Mai	207	545	752	62 (8.24)
Chiang Rai	482	1162	1644	82 (4.99)
Lamphun	191	489	680	32 (4.70)
Total	880	2196	3076	176 (5.72)

^a^ Report of Livestock numbers in Thailand 2019 by Information and Communication Technology Center, Department of Livestock Development, Ministry of Agriculture, Thailand.

**Table 2:** Molecular prevalence of *T. gondii* infection in goats according to different age groups and sexes of goats.

Factor	Category	Sample of Examined	Sample of Positive (%)	Chi-Square	df	*p*-Value
Age				2.981	1	0.0842
	≤2 years	167	12 (7.18)			
	>2 years	9	3 (33.33)			
Sex				0.1765	1	0.6744
	Female	165	15 (9.09)			
	Male	11	0			
	Total	176	15 (8.52)			

## Data Availability

The data sets supporting the results of this article have been submitted to GenBank, and the accession numbers are shown in the article.

## Supplementary Materials

The following supporting information can be downloaded at: https://www.mdpi.com/article/10.3390/vetsci12060555/s1, Figure S1: Sensitive species-specific primers as designed based on the complete double-strand sequence of a *Tgdhfr-ts* gene of pL0017 plasmid (MRA-786).

## Abbreviations

The following abbreviations are used in this manuscript:

DHFR-TS	dihydrofolate reductase thymidylate synthase
*Tgdhfr-ts*	*T. gondii* dihydrofolate reductase-thymidylate synthase
PCR	Polymerase chain reaction
