# Supplementary material for: Molecular Detection of Genetic Material of Toxoplasma gondii in Goat Blood Samples from Northern Thailand
Source: Vet Sci. 2025 Jun 5;12(6):555. doi: 10.3390/vetsci12060555 (PMC12197340; doi:10.3390/vetsci12060555)
Supplement: Supplementary file 1 [file vetsci-12-00555-s001.zip › vetsci-3649623-supplementary.pdf]

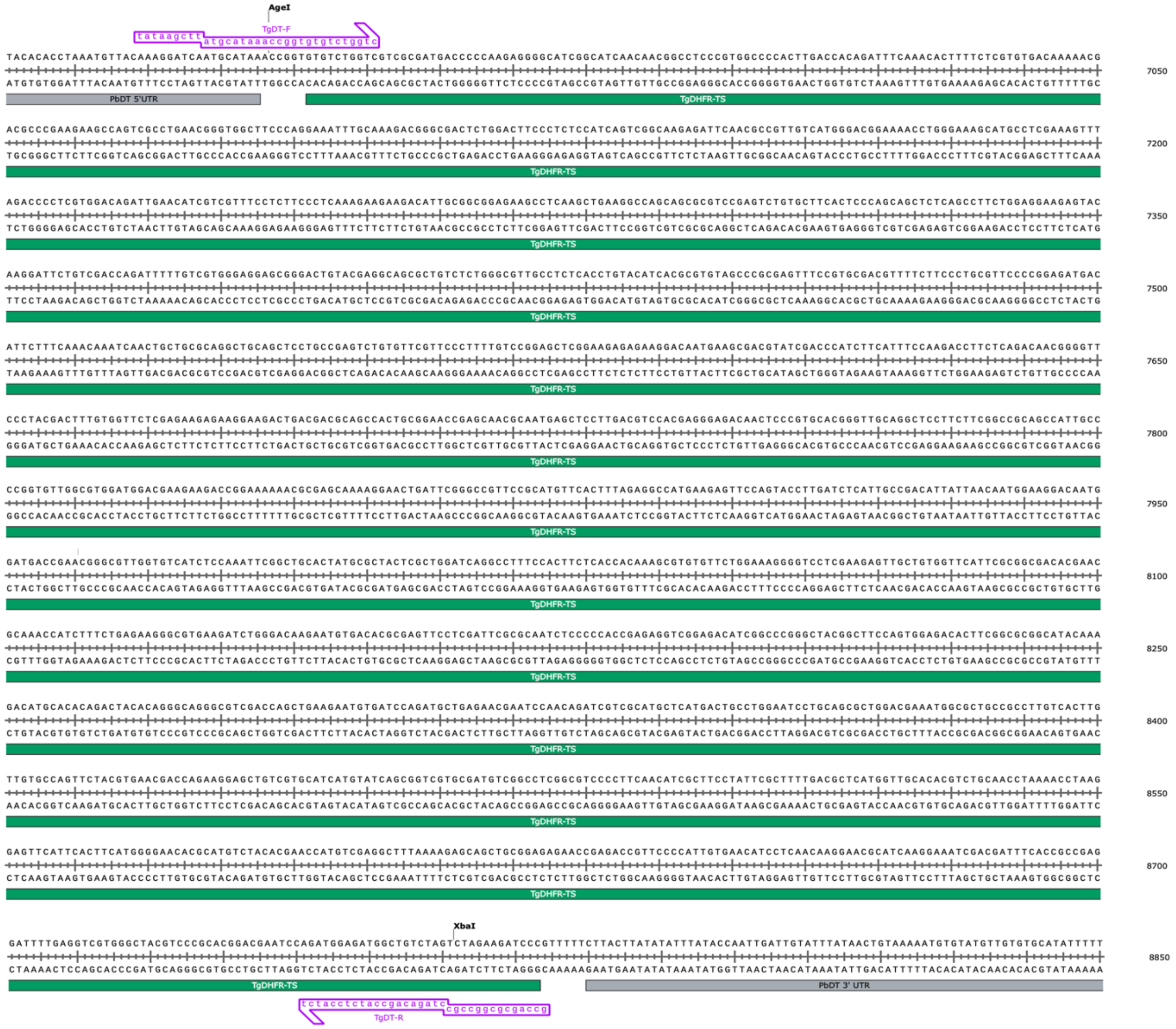

Figure S1. Sensitive species-specific primers as designed based on complete double strand sequence of a *Tgdhfr-ts* gene of pL0017 plasmid (MRA-786); TgDT-F is primer consisting of sense sequences and TgDT-R primer is an antisense sequence.
